# Challenges to the delivery and utilisation of child healthcare services: a qualitative study of the experiences of nurses and caregivers in a rural district in Ghana

**DOI:** 10.1186/s12912-024-01811-5

**Published:** 2024-03-14

**Authors:** Felix Kwasi Nyande, Esmeralda Ricks, Margaret Williams, Sihaam Jardien-Baboo

**Affiliations:** 1 Department of Nursing, School of Nursing and Midwifery, University of Health and Allied Sciences, Ho, Ghana; 2 Department of Nursing Science, Faculty of Health Sciences, Nelson Mandela University, Gqeberha, South Africa; 3 Faculty of Health Sciences, Nelson Mandela University, Gqeberha, South Africa

**Keywords:** Child healthcare, Nursing-related challenges, Health services utilisation, Inexperienced nurses, Shortage of nurses, Rural setting

## Abstract

**Background:**

Sub-Saharan Africa has one of the poorest child health outcomes in the world. Children born in this region face significant health challenges that jeopardise their proper growth and development. Even though the utilisation of child healthcare services safeguards the health of children, the challenges encountered by nurses in the delivery of services, and caregivers in the utilisation of these services, especially in rural areas, have contributed to poor child health outcomes in this region.

**Aim:**

This study explored the experiences of nurses and caregivers in respect of the nursing human resource challenges to the delivery and utilisation of child healthcare services in a rural district in Ghana.

**Methods:**

Individual qualitative interviews were conducted with ten nurses, who rendered child healthcare services; nine caregivers, who regularly utilised the available child healthcare services; and seven caregivers, who were not regular users of these services. These participants were purposively selected for the study. Data were collected using individual semi-structured interview guides and analysed qualitatively using content analysis. Themes and sub-themes were generated during the data analysis. The Ghana Health Service Research Ethics Review Committee and the Nelson Mandela University’s Research Ethics Committee approved the study protocol prior to data collection.

**Results:**

Three main themes emerged from the data analysis. Theme One focused on the shortage of nurses, which affected the quality and availability of child healthcare services. Theme Two focused on inexperienced nurses, who struggled to cope with the demands related to the delivery of child healthcare services. Theme Three focused on the undesirable attitude displayed by nurses, which discouraged caregivers from utilising child healthcare services.

**Conclusion:**

Nurses contribute significantly to the delivery of child healthcare services; hence, the inadequacies amongst nurses, in terms of staff numbers and nursing expertise, affect the quality and availability of child healthcare services. Also, caregivers’ perceptions of the quality of child healthcare services are based on the treatment they receive at the hands of nurses and other healthcare workers. In this respect, the bad attitude of nurses may disincentivise caregivers in terms of their utilisation of these services, as and when needed. There is an urgent need to comprehensively address these challenges to improve child healthcare outcomes in rural areas in Ghana. Relevant authorities should decentralise training workshops for nurses in rural areas to update their skills. Additionally, health facilities should institute proper orientation and mentoring systems to assist newly recruited nurses to acquire the requisite competences for the delivery of quality family-centred care child healthcare services.

## Background

Children born in Sub-Saharan Africa (SSA) face the steepest odds of survival due to preventable causes [1]. For these children, the risk of dying before the age of five is 12 times higher than that of their counterparts born in high-income countries [2]. Inadequate utilisation of child healthcare services due to service delivery challenges have contributed to the poor child health outcomes being experienced in SSA [3–5]. In Ghana, child health outcomes have been less than desirable particularly in rural areas, which is a source of concern [6]. The neonatal mortality rate in Ghana stands at 25 deaths per 1000 live births, while the infant and under-five mortality rates are 37 and 52 deaths per 1000 live births, respectively [7].

Globally, efforts are being made to attain the Sustainable Development Goal (SDG) 3 targets on neonatal and under-five mortalities. These targets seek to reduce neonatal and under-five mortalities to at least as low as 12 per 1,000 live births and 25 per 1,000 live births respectively, by the year 2030 [8]. Attainment of this target hinges on an availability of adequate human resource to effectively render child healthcare services. Globally, nurses constitute the majority of the healthcare workforce and are the backbone of primary healthcare service delivery [9]. Nurses form an essential component of the healthcare workforce as they play the crucial role of bridging the gap in health worker shortages, particularly in rural settings [6, 10]. Equity gaps still exist in the allocation of human resources in the health sector, including nurses, between rural and urban areas [6]. It is important to examine the impediments to progress, such as nurse-related challenges to the delivery and utilisation of child healthcare services in rural areas, which could derail the attainment of this goal.

Child healthcare delivery requires competent nurses to offer safe and holistic child healthcare services to patients and their families. Retaining essential health staff has been a significant challenge for low- and middle-income countries (LMICs), especially in rural areas [11]. Nurses working in rural areas in Ghana have described staff shortages as a major challenge to the provision of essential services [12]. These nurses have to multitask in the absence of adequate staff numbers, thus further compounding their workload and stress levels [13, 14]. Intricately linked to the shortage of nursing staff is the reality that inexperienced nurses must take up responsibilities for which they may not be prepared, therefore, they may be unable to provide supportive supervision and training to their subordinates [15]. Nurses working in paediatric facilities perceived the possession of practical experience to be related to a feeling of caring self-efficacy [16]. The lack of experience in the provision of specific child healthcare services has been reported as a concern for nurses [17, 18]. As the quality of nursing care declines, patient satisfaction is likely to plummet, because overburdened and inexperienced nurses are likely to perform poorly, which will lead to the eventual non-utilisation of healthcare services [19–21]. The interaction between healthcare workers and patients contributes to shaping caregiver perceptions of the quality of healthcare, and the continual utilisation of these services. Among underserved communities in SSA, the unfriendly attitudes of some healthcare workers have been a source of discouragement in the utilisation of child healthcare services [21, 22]. Client dissatisfaction with nursing care could result in their subsequent refusal to utilise the available child healthcare services [23, 24].

This study aimed to explore the views of both nurses and caregivers regarding the nursing human resource challenges experienced in the delivery and utilisation of child healthcare services in a rural district in Ghana.

## Methods

### Design

A qualitative approach, together with an exploratory and descriptive design, was used to conduct this study [25]. This enabled the researchers to gain insights into the meaning and experiences of the participants regarding the phenomenon under study within the context of a rural setting [26].

### Study setting

The study was conducted in selected healthcare facilities and communities within the Nkwanta South Municipality of the Oti Region, in Ghana. The municipality has two main hospitals, and several primary healthcare (PHC) clinics [27]. Six health facilities consisting of two hospitals, a health centre and three CHPS compounds were selected for the study. Healthcare service delivery at the district level in Ghana is organised into three: community, sub-district and district level. These facilities were purposively selected to include the different levels of service delivery at this level. Most of the population in this area live in rural settings, with nearly 41% of them having no formal education [27]. Additionally, eight communities were purposively selected and included in the study. These communities were selected with the assistance of the Municipal Health Directorate as they had high number of caregivers who did not utilise the available child healthcare services.

### Selection of participants

The study participants consisted of two groups: nurses, and caregivers with children under five years of age. These participants were purposively selected as they had the experience to answer the research questions [26]. The inclusion criteria for nurses were that they should be working at a public health facility within the municipality, and that they should have been directly engaged in the provision of child healthcare services for not less than six months prior to the commencement of data collection. Moreover, two groups of caregivers were included in the study: those who utilised the available child healthcare services, and those who did not. The inclusion of the two groups of caregivers enabled the researchers to explore the phenomenon from the perspectives of different caregivers. Those who utilised the available child healthcare services had to meet the following criteria: they should be taking care of a child who is less than five years of age; and attended any of the public health facilities to access child healthcare services at least twice within the past one year. Criteria for caregivers who did not utilise the available child healthcare services were that they should be a caregiver of a child who is less than five years of age; and have elected not to use child healthcare services within the past year even though there was a need to do so.

The nurse participants were sampled from health facilities that were purposively sampled to ensure adequate representation of nurses from the different locations within the municipality, and the different levels of the PHC system. The communities from which the caregivers were selected were purposively identified to be conterminous with the catchment areas of the nurse participants. With the assistance of the nurses and community health volunteers, the caregivers were identified and approached to participate in the study by the field investigator (FKN).

The ten nurse participants, and six of the caregivers who utilised the available child healthcare services, were selected from health facilities; the remaining ten caregivers were selected from the communities. The sample size of 26 participants was controlled by data saturation, wherein additional participants do not yield any new data, and the sample size was thus determined to be adequate [28].

Written permission was obtained from the Regional and Municipal Health Directorates, and the Municipal Assembly, before data collection commenced. In addition, permission was obtained from the management of the hospitals involved. The nurse participants were approached individually by the field investigator, whereas the field investigator was introduced by nurses to caregivers who consented to be interviewed. Caregivers who were interviewed in their homes were identified and introduced to the field investigator by nurses and Community Health Volunteers within those communities. The role of the caregivers in the study was explained to the head of each household, who then gave consent for their spouses to be interviewed. The caregivers were then approached and invited to take part in the study.

### Data collection

Data were collected from January to March 2019 through individual face-to-face interviews using semi-structured interview guides, designed for each participant category: nurses, caregivers who utilised the healthcare facilities, and caregivers who elected not to do so. All interviews were conducted by the field investigator. Prior to each interview, the interviewee and interviewer agreed upon a suitable date and time to minimise disruptions to the activities of study participants. Within the health facilities, nurses and caregivers were interviewed in open spaces, under trees located on the premises of the health facilities to avoid interruptions and to safeguard the privacy of the participants. In the communities, caregivers were interviewed either in a quiet corner or outside the main compound of their house, depending on which option provided a conducive atmosphere in which they could freely express themselves. A code was generated and assigned to each participant in order to associate them with a particular group in the study, while maintaining their anonymity.

Interviews with the nurse participants and four of the caregiver participants were conducted in the English language. The remaining caregiver participants were interviewed in the *Twi* a local Ghanaian language. The interviews were recorded using a digital audio recording device. Interviews with the nurse participants lasted between 45 and 60 min each, whereas the interviews with caregiver participants lasted approximately 30 min each. The audio recording of each interview was played back to the interviewee for the purpose of clarity and audibility, and for the interviewee to offer further clarification where necessary. The recordings were then transferred to the interviewer’s laptop for storage and processing. Fieldnotes made during the data collection process, were inserted into the interview transcripts prior to data analysis [25, 26].

### Data analysis

Data gathering and analysis were conducted simultaneously, as is practice in qualitative studies [29]. Data analysis was done using content analysis. The researchers applied the steps of qualitative data analysis, as outlined by Creswell [30]. After the interviews, the researchers engaged with the raw data by listening to audio recordings of interviews and renamed the audio files to reflect the participant codes. The audio recordings of interviews conducted in English were transcribed verbatim by the field investigator, with the help of two research assistants. Those interviews conducted in *Twi* were transcribed and translated into English by a language expert, to ensure that no meaning was lost in the process. The researchers immersed themselves in the data to make meaning of it, and then outlined their general impressions of the data [30]. Atlas.ti for Mac (version 8) was used to code and organise the data into categories.

In addition to the field investigator, an independent coder, experienced in qualitative data coding, coded the data applying the eight-step coding process described by Tesch [31]. After the initial engagement with the raw data, each interview transcript was read through carefully, several times over. Open and in vivo codes were created from a list of topics generated. These codes were then converted to appropriate axial codes using appropriate phrases. Clusters of similar topics were then formed into columns from which categories and themes were identified and matched with their appropriate descriptive topics.

### Ethical considerations

The study was granted ethical approval by the Research Ethics Committee of Nelson Mandela University (H18-HEA-NUR-018) and the Ethics Review Committee of the Ghana Health Service (GHS-ERC014/11/18). Each participant provided a written informed consent, prior to the collection of data. For participants who are illiterate, consent for participation in the study was obtained in the presence of a legal guardian. The study was explained to them in a language they understand, and legal guardian signed the consent form as a witness that the explanation was understood by the participant. All these were approved by the Research Ethics Committee of Nelson Mandela University (H18-HEA-NUR-018) and the Ethics Review Committee of the Ghana Health Service (GHS-ERC014/11/18). Participants were duly informed of their role, and of their right to withdraw from the study at any time, for any reason. Each participant’s right to privacy was upheld, as the interviews were held at locations agreed upon with the participants, and their responses were made anonymous to ensure confidentiality. The study adhered to all the requirements in the Protection of Personal Information Act, 2013 [32].

### Trustworthiness

To ensure the trustworthiness of the study, the criteria of credibility, dependability, transferability, and confirmability were used [26]. During the data collection process, the researchers ensured credibility by conscientiously following the interview guide and keeping to the subject matter of the interview, as part of reflexivity. Also, data from both nurses and caregivers were triangulated to capture the various perspectives of the phenomena under study. A thorough description of the entire research process was provided so that other researchers could replicate the study in similar settings or with similar participants, thus upholding transferability. Again, by defining the inclusion criteria, the researchers made sure that only participants who qualified by way of experience and location were recruited for the study. The researchers maintained a neutral point of view in the data analysis, as indicated by the inclusion of direct quotes from participants, to ensure confirmability. Verbatim transcriptions of data, together with the inclusion of direct quotations from participants were done to ensure that participants’ perspectives were adequately captured. Engaging an independent coder and audit trail were employed to ensure the dependability of the study.

## Results

### Demographic characteristics of participants

The study participants comprised ten nurses and nine caregivers who utilised the available child healthcare services, as well as seven caregivers who were not regular users of these services. The nurse participants comprised six females and four males, between the ages of 27 and 35 years. All the caregiver participants were females. Those who utilised the available child healthcare services were between 23 and 60 years old. Three of these caregivers had no formal education; four had attained secondary school education; one attained basic education; and one attained a tertiary education. Five of these caregivers were married, with two co-habiting with their partners. The seven caregivers who were not regular users of child healthcare services ranged from 29 to 47 years in age; three had no formal education, with the remaining four having either completed or dropped out at the basic school level. Five of the non-user caregiver participants were married, while one was single, and one was divorced.

Themes and sub-themes that emerged from the data analysis are depicted in Table 1.

**Table 1:** Themes and sub-themes from the data analysis

Theme	Sub-themes
1. Inadequate number of nurses to deliver quality child healthcare	1.1. Overburdened nurses 1.2. Too stressed to provide quality care 1.3. Reliance on auxiliary staff 1.4. Unavailability of services
2. Nurses inexperienced in child healthcare delivery	2.1. Newly qualified and inexperienced nurses 2.2. Learning on the job 2.3. Lack of regular in-service training
3. Undesirable attitude of nurses	3.1. Lack of sense of urgency 3.2. Poor in-patient care

#### Theme one: inadequate number of nurses to deliver quality child healthcare

Both nurse participants and caregiver participants revealed that the delivery of child healthcare services was confronted with a shortage of nursing staff, affecting the availability and quality of service delivery.

##### Sub-theme 1.1: overburdened nurses

The nurse participants indicated that the available staff did not match the number of patients who attended healthcare facilities to receive child healthcare services. Particularly, nurse participants in the hospitals were of the view that there were not enough nurses available to provide adequate care for the patients. The nurses appeared to be overwhelmed by the high proportion of total admissions that were made up of children, especially in the light of the few professional nurses available to provide the requisite services to these children. This is evidenced by the following quotes from selected participants:

*To admit over three thousand cases a year with just four staff nurses* (two ward managers), *and the rest are just assistants, means a lot.* (Hosp. Nurse 5)

*Yea, looking at our statistics here, the number of people who come on admission per year, if you are to group it in percentage-wise, about 40% are from children’s ward, so it tells that young ones do come to the hospital more than adults.* (Hosp. Nurse 1)

Nurse participants in the health centres and Community-based Health Planning and Services (CHPS) compounds, corroborated the assertion that there appears to be too many patients and shortage of nurses in the health facilities. They explained that the coverage areas of the clinics were too wide hence the increased patients attending the health facilities.

*Because the people are many. It’s not only* (this place); *some are from* (village A), *the other sides*, (Village B) *and the other places. They all come here. And the population too is more than the CHPS population.* (PHC Nurse 2)

The nurse participants described having to carry a heavy workload because of the high number of patients compared to the limited number of nurses. This caused them to experience feelings of tiredness and exhaustion. This experience was common to nurses at both the PHC clinics and the hospitals:

*… For instance, if the staff are enough, while you are admitting the child, another person will be laying the bed… but the staff are not enough so you finish the admission and go and lay the bed, come and set the IV cannula then you administer the drugs. But you see that is what we are doing here, doing three or four things at the same time.* (Hosp. Nurse 1)

*The workload is somehow tiring and heavy… On Tuesdays, when I do the CWC* [Child Welfare Clinic] *for some time, since they* (caregivers) *come in batches, I go inside and consult* (assess, diagnose and prescribe treatment for clients) *some people that have come for consultation. So, I just keep switching.* (PHC Nurse 3)

##### Sub-theme 1.2: too stressed to provide quality care

The nurse participants experienced significant stress, which would sometimes lead to a mental block as a consequence of the increased workload. The nurse participants also reported that they were unable to provide holistic quality care to their patients because of their heavy workloads.

*The workload is very stressful. It is very difficult and stressful.* (Hosp. Nurse 5)

*It puts a lot of pressure on the few of us* (nurses) *here. You can go to work, one person on a shift with about 16 neonates. How will you be able to render holistic care to these neonates?* (Hosp. Nurse 7)

Moreover, nurse participants who worked in the clinics explained that the combination of static clinic duties and outreach services placed increased pressure on them. Because they are overwhelmed by the number of patients who require their services at the same time, nurses are, at times, unable to fulfil all their responsibilities to their clients:

*In terms of child healthcare services, there is a big load, because we have to do outreaches every day of the week– and on Fridays, is our static clinic days. We have our static clinic on Fridays. That is why there is pressure.* (PHC Nurse 1)

*Under normal circumstances, the clients need counselling, but the pressure here doesn’t allow. You can’t even counsel the mother.* (PHC Nurse 3)

##### Sub-theme 1.3: reliance on auxiliary staff

In the hospitals, the auxiliary staff outnumber the professional nurses. These auxiliaries were seen performing nursing duties that were beyond their job descriptions on several occasions. According to the nurse participants, especially those based in the hospitals, because there were often more auxiliary staff than there were qualified nurses, they would, at times, delegate some of the nursing care to these auxiliaries, to reduce the workload. The auxiliaries filled the void left by the shortage of qualified nurses:

*We find ourselves in a place where the ward assistants and orderlies are more than the staff nurses. And they* (are expected to) *perform nursing activities…* [so you must equip them with the knowledge] *because they are not going back to school anymore; so that they can help more. Once they know what they are doing, it will help to decrease the workload.* (Hosp. Nurse 5)

*Sometimes, if we see that the registered nurses are not there, we may end up falling on the nurse assistants to be in-charge of a shift, if we have to, especially, in the night and sometimes in the afternoon.* (Hosp. Nurse 4)

Nonetheless, these sub-categories of staff do not have the requisite competences to perform certain nursing tasks. Thus, augmenting nursing staff strength with auxiliaries only increased the number of staff per head, but did little to reduce the workload on nurses:

*…you are the senior… and when you come to work with someone who doesn’t know anything, the whole pressure will be on you.* (Hosp. Nurse 2)

*Sometimes I can come to work with just ward assistants and almost everything that they do, you have to go ahead and supervise them.* (Hosp. Nurse 5)

##### Sub-theme 1.4: unavailability of services

The caregiver participants highlighted the shortage of nurses that affected the availability of child healthcare services, especially at the PHC clinics. The nurses often travelled for official assignments including attending meetings. They described occasions on which they tried to access child healthcare at PHC facilities but then realised that there were no nurses on site to attend to their sick children. Furthermore, those caregiver participants who did not utilise child healthcare services attributed their non-utilisation to the persistent absence of nurses at the PHC facilities:

*The reasons I don’t take my child to the hospital any time the child is sick are that, sometimes when you take the sick child to the clinic, you don’t get health professionals to attend to the child.* (Non-user 5)

*This child, for instance, when he experiences stomach upset right now, I don’t know what to do for him, so I’ll run to the facility for treatment but by the time I get there, I won’t meet anybody there.* (Non-user 3)

The caregiver participants who did not utilise the available child healthcare services blamed the persistent absence of nurses at the respective health facilities for their non-utilisation. According to them, the situation left them with no choice but to turn to other available options, such as over-the-counter self-medication:

*In such instances, you have no option but to return home with your sick child.…So, when that* (absence of nurses) *happens I go to the drug store to get the drugs so that I can give to him.* (Non-user 1)

#### Theme two: nurses inexperienced in child healthcare delivery

Interviews with nurse participants revealed that the nurses were not adequately skilled to deliver child healthcare services. Most of them lacked training in the provision of care to sick children. They thus resorted to learning on the job.

##### Sub-theme 2.1: newly qualified and inexperienced nurses

Interviews with the nurse participants revealed that many of the staff assigned to the children’s units of hospitals were newly qualified with very little nursing experience, let alone in child healthcare which requires specialised competences. These nurses were unable to perform the tasks required of them:

*Most of the staff, it is after school or national service, you can see that their experience is a bit limited because this is more or less like a special ward because of the children.* (Hosp. Nurse 4)

These nurses felt inadequately skilled to render quality care to sick children under their care. Even nurses who had been working in this area for some time questioned their own expertise in managing paediatric cases under their care. Their self-doubt was quite evident in the narratives:

*…at this place, we do most of the work by ourselves and the question is: “is it really the correct thing we are doing or not?”* (Hosp. Nurse 6).

*Even myself, I can’t say that I am adequately equipped. And if I am not…you will definitely know that my subordinates too may not be properly equipped.* (Hosp. Nurse 4)

Interestingly, some nurse participants indicated that they often found themselves in situations in which they had to perform tasks which they did not have the requisite expertise to perform correctly. In such instances, they were expected to gather the confidence to perform these tasks since there was nobody else to do so:

*When you meet the case and there is no one there, you have to do it by yourself. No matter what happens, you have to gather the confidence and do it, whether what you are doing is right or wrong.* (Hosp. Nurse 5)

*In my field, as a paediatric nurse, maybe with the breath sounds which I am not very conversant with, I need a prescriber, so that when I assess a child that I hear crackles, the person should be able to confirm that what I heard are really crackles and not maybe a wheeze or something else* (Hosp. Nurse 6).

The PHC facilities are the gatekeepers to the healthcare system in Ghana. However, because they are experienced in attending to sick children, nurses in these facilities would just refer the sick child to the next level, without any attempts to stabilise them. One nurse narrates her experience as follows:

*For me, if I am not sure of something I just refer* [to the district hospital]. (PHC Nurse 2)

The lack of experience in the delivery of child healthcare services contributed to caregivers losing trust in the PHC clinics and side-stepping them to higher level facilities. This situation troubled some of the nurse participants as it could lead to a disruption in their role as gatekeepers in the PHC system:

*They* (nurses PHC facilities) *don’t know anything, so sometimes the* [patient’s] *relatives see the* [PHC] *facilities as useless. They don’t even go there at all. They don’t think they can offer them anything. They feel if they go there, they will be given referral letter to go to the hospital, so they end up not going there at all.* (Hosp. Nurse 4)

##### Sub-theme 2.2: learning on the job

Nurse participants indicated that they had to devise various strategies to gain experience in the provision of child healthcare. They either learned using the cases with which they are confronted, or they sought support from their experienced colleagues through social media channels.

Some of the nurse participants indicated that they gained confidence over time, from performing a particular task on numerous occasions. Others indicated that they learned most of their child healthcare skills from experienced nurses and midwives with whom they worked.

*It was difficult* (initially). *Something you have not done before, you are now coming to do it, definitely it will take you some time… When I came* (to the paediatric ward), *like setting* (IV lines), *you know public health* (nurses), *we don’t enter the vein, you go IM.* (Hosp. Nurse 1)

*Where I was working at first, we had a midwife [that] I was learning that* (child healthcare nursing) *from. I don’t even know if it’s everything I learnt.* (PHC Nurse 2)

Again, some of the nurses resorted to sharing their challenges and getting help from their colleagues using a social media platform (e.g. WhatsApp) when they were not sure of how to care for sick children:

*For my ward, we have a platform. The idea for the platform is that, if you are managing a case and you are stranded, and because we don’t have the doctors on the ward, you can put it on the platform. Anybody who has knowledge about will do this thing.* (Hosp. Nurse 6)

##### Sub-theme 2.3: lack of regular in-service training

Routine professional development training is required to improve the competences of practicing professionals; however, the nurse participants lamented the lack of routine workshops in child healthcare. They believed this situation contributed to the difficulties that encountered in their work.

The experienced staff in the child healthcare units were thus compelled to train newly qualified nurses in specific aspects of child healthcare delivery:

*There has not been anything like in-service training for paediatric nurses. Nothing like that!* (Hosp. Nurse 5)

*We end up having to train them* (new staff) *in paediatric assessment.… and also paediatric medication calculations, because we do a lot of calculations for the children, and how to avoid medication error in administering medication to the children.* (Hosp. Nurse 4)

The nurse participants expressed concern that the scheduling of in-service training was not regular, and that it was not well publicised. They claimed that this situation affected their competency in, and ability to keep abreast of, developments in child healthcare:

*Since I have been here, there’s not been any training in paediatrics… It is not frequent at all, for over two*, (or) *three years now we have not even heard of anything about paediatrics or something for you to see how you can equip yourself.* (Hosp. Nurse 4)

*It was only once that I went for refresher training. About two years ago. You see, health is dynamic, maybe every six months we need to undergo such training sessions. Maybe you think what you are doing is right, but something has been added to it, or something has been subtracted from it.* (PHC Nurse 1)

The lack of in-service training negatively impacted the work of nurses. Moreover, it contributed to the pressure felt by the nurse managers, who had to manage staff that were not competent in the performance of their duties. The nurse participants indicated that they had to devise ways to keep themselves updated, while also training their subordinates:

*It* (lack of in-service training) *really affects our work, and it gives a lot of pressure on the unit manager. It means that you have to find a way to develop yourself first and be able to help your subordinates too. Other than that, all of you will be archaic in the system.* (Hosp. Nurse 4)

##### Theme three: undesirable attitude of nurses

The attitudes that nurses display towards caregivers, and their sick children, was raised as a concern amongst caregivers. Both nurse and caregiver participants mentioned nurses’ attitudes and behaviours as undesirable and unprofessional.

#### Sub-theme 3.1: lack of sense of urgency

Caregivers described being displeased with the attitude of nurses towards the care of their sick children and highlighted that this required urgent attention. They believed the nurses did not attach any sense of urgency to their work, even in cases of emergency.

*The nurses are our main problem. When your child is sick, and you go and tell them, they won’t mind* [attend to] *you.* (Caregiver 9)

*Sometimes too, when you get to the hospital, the doctors and nurses will be in the rooms and will not attend to you immediately but will behave as though they don’t care until the time they like…*[nurses] *behaving unconcerned and remaining in their houses even when patients are in pain and waiting for their services. That attitude is not good.* (Non-user 5)

The caregiver participants complained about attitude and behaviour of the nursing staff at the PHC facilities. They expressed misgivings that such behaviour contributed to limited access to child healthcare services:

*We can say we have a health facility here so that when something happens to you can run to them for treatment, but they are not available for us on any day. So, we are suffering here.* (Caregiver 9)

*They* (nurses) *don’t take good care of the patients because they don’t show compassion towards us the clients, that visit the facility.* (Non-user 4)

The PHC clinics have working hours outside which they remain closed. After the closing hours, the facilities turned away patients, irrespective of the urgency. This situation was a matter for concern to caregiver participants:

*You see sometimes you may take a sick child to the health facility at dawn, then the health workers will tell you that it is too early for them to attend to you. Meanwhile, the person is also suffering. So, I will take the person to a place that they can take care of him/her, even if it is early in the morning.* (Non-user 4)

##### Sub-theme 3.2: poor in-patient care

Caregiver participants described their experiences with various aspects of in-patient nursing care that they felt fell short of their expectations.

The caregiver participants indicated that, to a large extent, the nurses left certain aspects– related to the care of sick children– to the caregivers to perform. However, nurses failed to supervise the caregivers in the performance of these duties:

*Like some nurses it is their duty to wake up at 5am to give medication to your child but they won’t do it; they will rather ask you to do it. The person will just tell you to pour it to this level and go– they won’t wait to look at the level you have poured it to. Instead of pouring the medicine to the right level and giving it to you to be given to the child, some will not do it.* (Caregiver 4)

*For the bed sheets, they are not changing it when they give you one it is only one till the day they will discharge you. You will have to remove to it and go and wash it and bring it back. It is not good, but we manage it.* (Caregiver 5)

Caregiver participants also found communication between nurses and caregivers to be worrisome. According to the caregivers, both verbal and non-verbal communication by nurses fell short of their expectations. The attitude that nurses expressed through their body language was described as hostile:

*She* (the nurse) *was harsh. I don’t know how to describe it… She did not say any bad words to us but her actions and body language. You could see that she is not friendly.* (Caregiver 2)

*Sometimes you would find them* (nurses) *speaking in a language I don’t understand with an unfriendly expression on their faces. It feels like they are talking about you. Other times too, you would get to the facility with the sick child convulsing, sitting in front of a nurse, I greet and she responds but she doesn’t say anything else or do anything else, she seems busy on her phone.* (Caregiver 8)

Furthermore, both nurse and caregiver participants described verbal utterances from some of the nurses as unpleasant. Caregivers complained about the manner of communication displayed by nurses, which they felt did not convey respect. According to the caregivers, communication between nurses and clients was sometimes characterised by the nurses yelling and casting invectives at caregivers. Caregivers felt dehumanised by the utterances made by nurses.

*At times, too, the way some of the nurses talk to us patients and relatives is not good, as we are human beings; that one too will make you feel bad.… The person* (nurse) *just sees what you are doing once and then you see that she* (nurse) *becomes annoyed and starts talking… shouting.* (Caregiver 5)

*At times, when you talk to them* (the nurses), *they shout at you for asking them to repeat what they said to you. They ask you what were you listening to when they spoke to you?* (Non-user 4)

Although they felt terrified, angered, and alarmed by the attitude displayed by the nurses, they had to put up with these unwelcoming attitudes for their sick children to be attended to. Moreover, for fear of being victimised, the caregivers tried to avoid getting into confrontations with the nurses. Some of the caregiver participants described how they muddled through the unprofessional and uncaring behaviour and attitudes of nurses:

*It hurts. I feel anger in me but, since it’s my child who is sick and needs medical attention, I wouldn’t want to offend the nurses. So, because of that, I stay calm.* (Caregiver 8)

*Many, at times, you tell yourself that if not because of the sickness, like, I wouldn’t have been here, so I have to manage it like that.* (Caregiver 5)

Both nurse and caregiver participants realised that unfriendly attitude towards caregivers could discourage the utilisation of child healthcare services. The participants described the negative encounters with nurses as a disincentive to utilising child healthcare services:

*But some* [of the nurses], *the way they will treat you, it will even drive you away from the hospital.* (Caregiver 5)

*The attitude of some of the nurses can sack clients from coming to the hospital. If my child is sick and I come to you and all that you are doing is pressing phone, and the child’s condition is getting worse, will I come tomorrow again?* (Hosp. Nurse 2)

## Discussion

The results of the study revealed that nurse participants felt overwhelmed and over-worked by their daily activities. Care of children requires continuous contact with patients and family members who are facing critical situations; this places a high demand on nurses and exposes them to significant psychosocial risks and burnout [33]. This finding is consistent with previous studies conducted in rural communities in SSA where nurses described being overwhelmed with work because of inadequate staffing [5, 12, 13].

The few available nurses were thus compelled to multi-task to cope with the excessive workload. This however, stressed them further, and could compromise the quality of child healthcare services. These findings are congruent with those of previous studies in rural settings, in LMICs, which determined that nurses often multi-tasked in the face of staff shortages [14, 34]. The mismatch between nursing staff strength and workload has been found to be correlated with poor quality nursing care [14, 19, 20, 35]. As the quality of nursing care falls, patient satisfaction is likely to plummet, which will lead to the eventual non-utilisation of healthcare services.

Our study also found nurses delegated some of their nursing responsibilities to the auxiliaries. Similar findings have been reported in Ghana and Kenya, where nursing staff shortages are also rampant [12, 36]. However, deploying auxiliaries to render nursing care did little to ameliorate the workload of nurses, as the nurses ended up spending extra time ensuring that the auxiliaries performed the relevant tasks correctly. In addition, placing auxiliary staff in charge of shifts could greatly compromise nursing care during those shifts. This situation would lead to decreased quality of care, and to patient dissatisfaction. Previous studies have consistently made similar findings in this regard [37, 38]. As caregivers become dissatisfied with nursing, they are more likely to discontinue their utilisation of the available services.

Access to child healthcare services was found to be affected by the unavailability of nurses and limited operational hours, particularly at the PHC clinics. Efforts by caregivers to access child healthcare services for their sick children was often thwarted by the persistent absence of healthcare providers at clinics. Previous studies have also found the unavailability of healthcare workers to be cause of the concomitant unavailability of child healthcare services [39–41]. Similarly, in resource-constrained settings, the limited operational hours of primary healthcare facilities have been reported to affect the availability and utilisation of healthcare services [21, 42]. To overcome these challenges, caregivers relied on the services of drug sellers and other means of treating their sick children. This is also consistent with previous studies [39].

The second theme of the study highlighted the fact that nurses were inexperienced in the delivery of child healthcare services. These nurses were newly qualified and had to learn to provide child healthcare services ‘on the job’. This situation was worsened by the absence of continuous professional development training to build the capacity of nursing staff. By questioning their own competences in the performance of certain tasks, nurse participants subtly agreed that the quality of child healthcare services offered was not the best. Having to perform unfamiliar procedures and undertake tasks that they were uncertain of, without the necessary supervision, could result in harm to patients. These findings support those of previous studies [13, 15, 18] all of which reveal that a lack of practical experience correlated with feelings of inadequacy in the provision of care to paediatric patients.

The recent demographic shift in the nursing population in Ghana is the result of an increase in the number of nurses churned out from training institutions, thus causing an influx of inexperienced nurses into the healthcare system [43]. The shortage of nursing staff has caused an increase in the training and recruitment of fresh graduates from nursing schools; however, these graduates do not have the mentorship of experienced nurses available to them. These newly trained nurses require the mentorship of experienced nurses for them to fit into a specialised area, such as child healthcare. Coupled with the lack of a properly instituted orientation and in-service training programme, especially for newly qualified personnel, this could lead to the delivery of poor-quality care. Previous studies identified similar findings that health workers serving in rural communities complained of the lack of training opportunities and mentorship [14, 44]. Continuous professional development and in-service training helps nurses keep abreast of current trends and changes in healthcare delivery. The lack of regular refresher courses in one’s area of practice could result in the professional becoming obsolete, over time.

Interactions between healthcare workers and patients contribute significantly to shaping caregiver perceptions of the quality of healthcare available to them. Caregivers complained about the attitude of nurses, which they described as unsatisfactory. This discouraged caregivers from utilising child healthcare services. Similar findings related to bad attitudes on the part of healthcare workers, which caused patients and relatives to be unhappy with their utilisation of healthcare facilities, have been highlighted by previous studies [3, 23, 24, 45]. The negative experiences of caregivers remain worrisome, as these could contribute to current users becoming non-users, thus further worsening the state of child health in the Nkwanta South Municipality. Current non-users were once users who have since discontinued their use of these healthcare services because of the perceived bad attitudes of nurses. The acceptability of healthcare services is an inclination to utilise healthcare services. In this respect, for universal coverage to be achieved, the available healthcare services must be found to be acceptable by the people who need it, when they need it [46]. Their unwillingness to utilise the services provided by health facilities could be the populace’s way of protesting the unacceptability of the services rendered to them.

## Limitations

The study was limited to only nurses and caregivers of children under five years of age. The perspectives of other important stakeholders such as the Municipal Health Directorate, managers of the two hospitals, and other healthcare workers were not assessed. Additionally, the perspectives of the families of the caregivers were not assessed. The perspectives of these other stakeholders could be explored in future studies. Also, the extent and impact of the nursing human resource constraints on the overall child healthcare outcomes could not be assessed because of the qualitative nature of the study. Future studies may consider assessing quantitatively, the impact of these challenges on overall child healthcare delivery and outcomes.

## Conclusions

The delivery of quality child healthcare and the timely utilisation of these services contribute to improved child health outcomes. Nurses constitute most of the essential healthcare human resources; hence, inadequacies of nursing staff– in terms of numbers and expertise– also affect the quality of child healthcare services. Additionally, caregivers form their own perceptions about the quality of available services based on the treatment they receive at the hands of nurses and other healthcare workers. Thus, the bad attitude of nurses could serve as a disincentive to caregivers’ utilisation of these services. There is the need to comprehensively address these challenges in order to improve child healthcare outcomes in rural areas.

Furthermore, there is a need to reconsider current policy guidelines on the recruitment of nurses to augment staffing numbers in rural areas, with particular emphasis on the distribution of nurses with expertise in child healthcare. In addition, the continuous professional development training of nurses involved in child healthcare delivery will further equip them to deliver quality child healthcare services. These training workshops should be decentralised and made accessible to nurses in rural areas, so that they can also benefit from such initiatives. Health facilities should institute proper orientation and mentoring systems, as well as customer care training that would assist nurses to acquire the requisite competences for the delivery of quality family-centred care child healthcare services.

## Data Availability

The datasets for the study are not publicly available; this is due to the study’s ethical requirements to ensure respondent anonymity in reporting, and confidentiality in participating in the study. These datasets are, however, available from the corresponding author, upon reasonable request.
